# Sparse and stereotyped encoding implicates a core glomerulus for ant alarm behavior

**DOI:** 10.1016/j.cell.2023.07.022

**Published:** 2023-08-03

**Authors:** Taylor Hart, Dominic D. Frank, Lindsey E. Lopes, Leonora Olivos-Cisneros, Kip D. Lacy, Waring Trible, Amelia Ritger, Stephany Valdés-Rodríguez, Daniel J.C. Kronauer

It has come to our attention that there was an error in the concentration of injected plasmids reported in this Article. In Table 1, column 1, the unit of the injected plasmid DNA concentration is incorrectly given as “pmol/μL.” The correct unit is “fmol/μL.” This error was repeated in the Method Details section on “Egg collection, microinjection, and larval rearing” (page e5, paragraph 2, third line). The units have been corrected in the article online. The authors apologize for the error.

